# Functional performance and patient satisfaction comparison between a 3D printed and a standard transradial prosthesis: a case report

**DOI:** 10.1186/s12938-022-00977-w

**Published:** 2022-01-29

**Authors:** Christopher Copeland, Claudia Cortes Reyes, Jean L. Peck, Rakesh Srivastava, Jorge M. Zuniga

**Affiliations:** 1 Department of Biomechanics, University of Nebraska at Omaha, Biomechanics Research Building, 3D Printed Prosthetic,Orthotic & Assistive Devices, Omaha, USA; 2 CHI Health Creighton University Medical Center and an Adjunct Faculty at the Department of Occupational Therapy at Creighton University, Omaha, USA; 3 Innovative Prosthetics & Orthotics, Omaha, USA

**Keywords:** Transradial prosthesis, Computer-aided design, 3D printing, Transitional prosthesis, Functional outcome, Patient satisfaction

## Abstract

**Background:**

The delay between amputation and prosthesis fitting contributes to the high rate of prosthetic abandonment despite advances in technology. Three-dimensional (3D) printing has allowed for the rapid fabrication of prostheses. Allowing individuals with amputations to interact with a prosthesis shortly after their procedure may reduce rejection chances. The purpose of the current investigation is to compare functional outcomes and patient satisfaction between a standard transradial prosthesis fitted in a clinic with a 3D-printed prosthesis fitted remotely. The standard prosthesis featured a hook terminal device, while the 3D printed prosthesis’ terminal device was a functional hand.

**Results:**

The main finding of this case study was that the use of a 3D printed arm prosthesis fitted remotely resulted in better functional performance, but lower overall patient satisfaction than the standard arm prosthesis. Use of the 3D printed arm resulted in improved gross manual dexterity as measured by the Box and Block test. The 3D printed prosthesis also allowed improved performance in bimanual coordination. However, the standard-hook device scored higher in patient satisfaction survey results. The patient's concerns with the 3D printed prosthesis were the durability and effectiveness of the device.

**Conclusion:**

While durability and complex grip patterns remain a concern, the positive attributes of 3D printed prostheses include visual appeal, ease of donning, and customization of parameters to improve upper-limb symmetry offers a promising option to familiarize new amputee patients with the use of a prosthesis. Rapid manufacturing and remote fitting allows 3D printed devices to serve as postoperative transitional devices and may function as definitive devices with minimal loss of functionality if standard clinic-based prostheses are not available.

**Methods:**

The patient was a 59-year-old male with a traumatic transradial amputation of the dominant arm. A 3D printed transradial prosthesis was remotely fitted and manufactured using photogrammetry. Assessments were performed initially with the standard-hook prosthesis and then with the 3D printed device after a 5-week familiarization period. Functional outcomes were evaluated using the Box and Block Test and Bimanual Coordination Tray Test. Patient satisfaction was evaluated using two self-reported questionnaires (the QUEST 2.0 and the modified OPUS).

## Background

By the year 2050, an estimated 3.6 million persons will be living with amputations within the United States [1]. In the fiscal year 2016, 22% (*n* = 20,158) of the veterans who received amputation care at Veterans Affairs medical facilities had experienced an upper-limb amputation [2]. Of those, 16% (*n* = 3225) had a major upper-limb amputation. Furthermore, according to the National Trauma Databank [3], 0.09% of persons hospitalized after trauma sustained a major upper-limb amputation. The most common upper-limb amputation seen in adults is at the transradial level [4]. Despite advances in upper-limb prostheses, there continues to have a high rate of user abandonment [4]. Up to 52% of upper-limb amputees rejected or abandoned their prosthesis [2].

Fitting a patient with a prosthesis within 4 weeks after amputation will increase the likelihood of acceptance of the device [5]. This time is known as the 'golden period' of upper extremity prosthetic rehabilitation and may be the most vital factor in the patients' acceptance of the prosthesis [6]. During this period, contractures and muscle atrophy are common risk factors that can affect prosthesis use and overall function. Preserved range of motion and strengthening of the affected musculature is crucial in maintaining body symmetry, posture, and alignment of the upper quadrant to reduce overuse injuries [7]. The use of transitional prostheses decreases the burden on the contralateral limb, increases function by offering an additional grasp with bimanual tasks, assists in body symmetry, and improves self-image [8]. It has been reported that immediate postoperative prosthesis use (i.e., transitional prosthesis) can also improve the range of motion and strength of the affected limb [9]. However, these transitional devices are often made by hand, requiring long construction time and highly skilled technicians to manufacture them [9, 10].

The ability to develop low-cost, lightweight, customized and user-friendly upper-limb transitional prostheses designed to familiarize the transradial amputee patient with the use and function of different prostheses can have a significant impact in the reduction of prosthetic rejection of a definitive prosthesis [11]. Recent technological advances in computer-aided design programs and additive manufacturing, otherwise known as three-dimensional (3D) printing, offer the unique possibility of fitting, designing, and manufacturing low-cost and customized upper-limb 3D printed prostheses remotely [7, 12, 13]. While their rapid production and low cost has been documented and suggests 3D printed devices can serve as transitional devices, little literature has investigated their functionality compared to a common clinic-based prosthesis, the prosthetic hook (henceforth: standard prosthesis). Thus, the purpose of the current investigation is to compare functional outcomes and patient satisfaction between a standard transradial prosthesis fitted in a clinic with a 3D printed arm prosthesis fitted remotely. This information is crucial for the implementation of 3D printed prostheses as transitional prostheses, or definitive prostheses in areas without access to standard prostheses from a clinic. We hypothesized that there would be no significant differences for functional or patient satisfaction outcomes between a standard arm prosthesis and a 3D arm printed prosthesis. Our hypothesis is based on previous investigations that have described the functionality of 3D printed prostheses [14] and their potential use as transitional prostheses [7, 12].

## Results

**Fig. 1:**
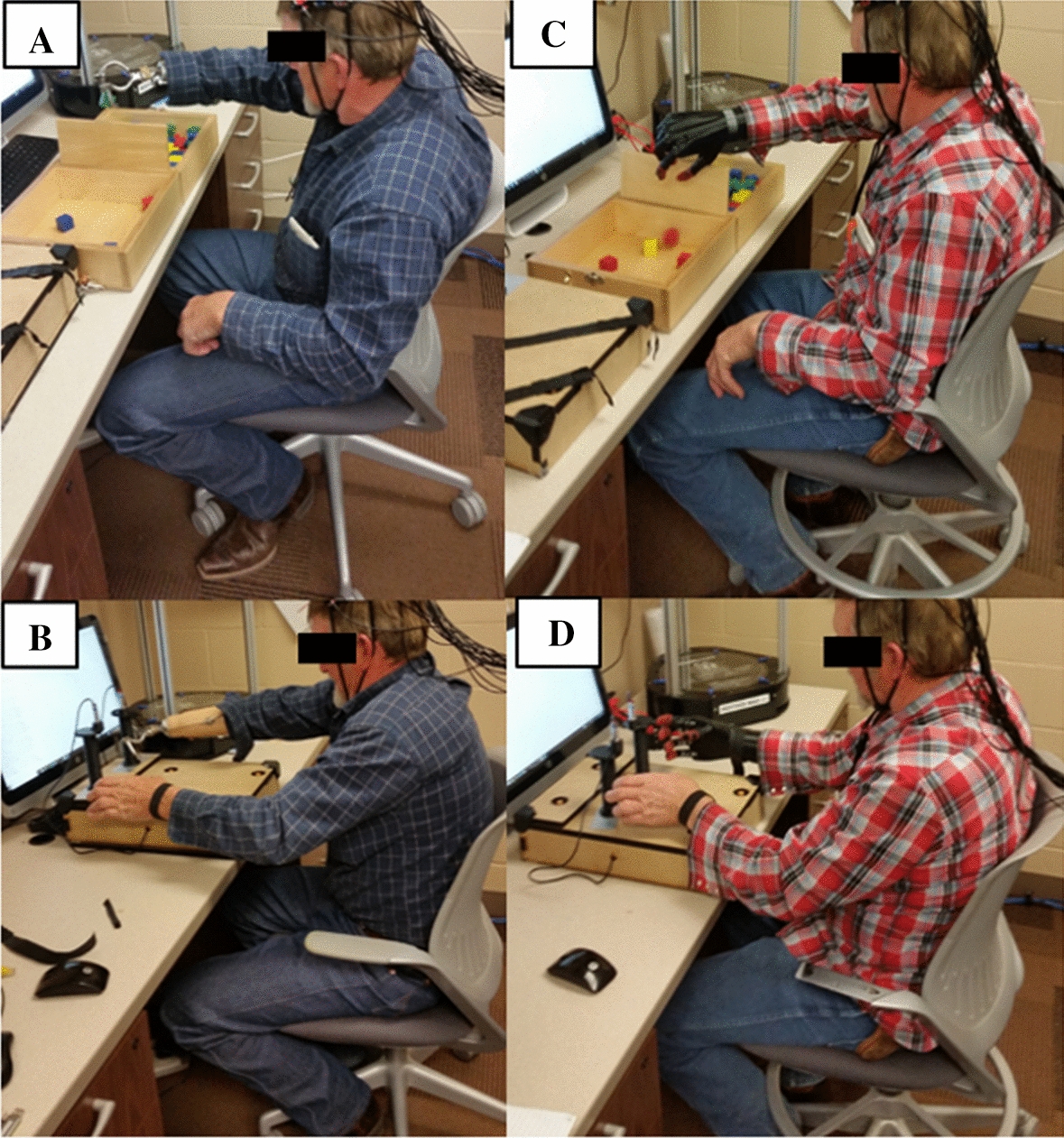
Functional testing. **A** Box and Block Test with the standard prosthesis and the 3D printed arm prosthesis, respectively. **B** Bimanual Coordination Tray Test with the standard prosthesis and the 3D printed arm prosthesis, respectively. The functional testing was performed after using each prosthesis for 4 weeks. **C** Bimanual coordination with the standard prosthesis. **D** Bimanual coordination with the 3D printed arm prosthesis

**Fig. 2:**
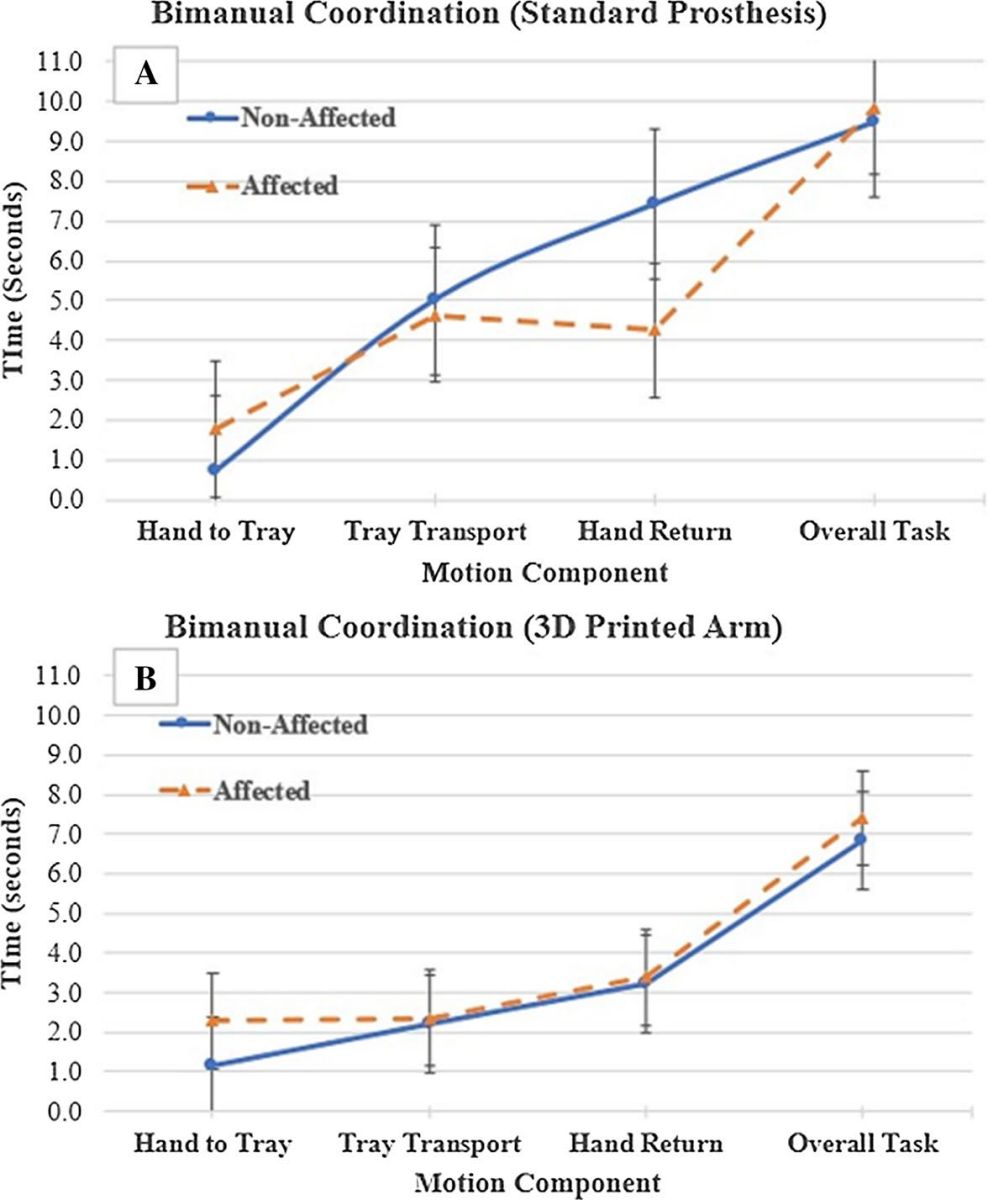
The bimanual task required the participant to grasp and transport a tray to assess synchrony of the upper limbs during a functional reach-to-grasp bimanual task. Use of the standard prosthesis during the task (**A**), the participant displayed an observable but statistically insignificant (Student’s t-test, *p* = 0.29) asynchrony while returning his hands to the starting position using the standard prosthesis. This trend of asynchrony was not observed during the use of the 3D printed arm (**B**)

Table 1 summarizes the functional assessments for the Box and Block Test (Fig. 1A, C) and the Bimanual Coordination Tray (Fig. 1B, D) for the first visit using the standard arm prosthesis and the second visit using the 3D printed arm prosthesis. The participant performed significantly better (Student’s *t*-test, *p* < 0.05) using the 3D printed arm prosthesis (17.0 ± 1.0) compared to the standard prosthesis (12.3 ± 1.2) in the Box and Block test. In terms of bimanual coordination, the participant displayed non-significant differences in overall temporal coordination between using the 3D printed or standard arm prostheses. However, there appears to be an observable but insignificant difference (Student’s *t*-test, *p* = 0.29) in hand-return coordination when using the standard prosthesis, compared to the 3D (Fig. 2). The QUEST questionnaire's descriptive information for the standard and 3D printed arm prostheses are summarized in Table 2. The OPUS satisfaction and functional items for the standard and 3D printed arm prostheses are summarized in Tables 3 and 4.

**Table 1:** Functional assessments

	Box and blocks (blocks per minute)				Bimanual coordination tray (overall time in seconds)			
	Trial 1	Trial 2	Trial 3	Mean ± SD	Trial 1	Trial 2	Trial 3	Mean ± SD
Visit 1								
Standard Prosthesis	13	11	13	12.3 ± 1.2	14.15	7.34	8.10	9.86 ± 3.73
Non-affected hand	43	50	53	48.7 ± 5.1	14.75	6.87	6.84	9.49 ± 4.56
Visit 2								
3D printed arm	16	17	18	17.0 ± 1.0*	7.98	7.07	7.17	7.41 ± 0.50
Non-affected hand	42	44	43	43.0 ± 1.0	7.64	6.17	6.71	6.84 ± 0.74

*Student’s *t*-test, significantly greater mean difference (*p* ≤ 0.05).

**Table 2:** Quebec user evaluation of satisfaction with assistive technology (QUEST) ratings

Items			3D printed arm		Standard prosthesis	
How satisfied are you with:						
Dimensions (size, height, length, width)			4		3	
Weight			4		4	
Adjustments (fixing, fastening)			3		4	
Safety (secure)			4		4	
Durability (endurance, resistance to wear)			2		5	
Ease of use			4		5	
Comfort			3		4	
Effectiveness (the degree to which your device meets your needs)			2		5	
Device satisfaction			3.25		4.25	

Services	3D printed arm	Standard prosthesis
How satisfied are you with:		
Service delivery	3	4
Repairs and servicing	3	4
Professional services	4	4
Follow-up service	4	4
Service satisfaction	3.5	4

1 = not satisfied at all, 2 = not very satisfied, 3 = more or less satisfied, 4 = quite satisfied, 5 = very satisfied

**Table 3:** Orthotics Prosthetics Users Survey (OPUS) frequency distribution for satisfaction items

OPUS Module 5	Raw scores		
Satisfaction items	3D Printed arm		Standard prosthesis
My prosthesis fits well	4		4
The weight of my prosthesis is manageable	5		4
My prosthesis is comfortable throughout the day	4		4
It is easy to put on my prosthesis	5		4
My prosthesis looks good	5		4
My prosthesis is durable	4		5
My skin is free of abrasions and irritations	5		2
My prosthesis is pain-free to wear	4		4
My clothes are free of wear and tear from my prosthesis	4		2
I can afford the out-of-pocket expenses to purchase and maintain my prosthesis	3		3
I can afford to repair or replace my prosthesis as soon as needed	3		4
Average	4.2 ± 0.75		3.6 ± 0.92

5 = strongly agree, 4 = agree, 3 = neither agree nor disagree, 2 = disagree, 1 = strongly disagree, 0 = don't know. Mean values calculated excluding 0 = don't know raw scores

**Table 4:** Orthotics Prosthetics User Survey (OPUS) frequency distribution for functional items

OPUS Module 3	Raw scores	
Functional items	3D printed arm	Standard prosthesis
Drink from a paper cup	4	4
Use fork or spoon	3	4
Pour from a 12 oz. can	3	4
Dial a touch-tone phone	3	4
Stir in a bowl	3	4
Put on and take off prosthesis	4	4
Average	3.3 ± 0.52	4.0 ± 0.0

5 = very easy, 4 = easy, 3 = slightly difficult, 2 = very difficult, 1 = cannot perform activity, 0 = not applicable. Mean values calculated excluding 0 = not applicable raw scores

The self-reported surveys, however, indicated that overall the research participant seemed more satisfied with the standard arm prosthesis than the 3D printed arm. The 3D printed prosthesis was superior for items related to the prosthesis dimensions (such as size and manageable weight; Tables 2, 3), ease of donning and doffing, and esthetics of the device (Table 3). The surveys showed that the research participant had a consistent concern with the durability of the 3D printed arm (Tables 2, 3), although no failures were reported. In terms of reported function, both prostheses allowed the research participant to perform activities that use gross or composite grasping, such as drinking from a paper cup (Table 4). However, for activities that require individual finger movement or different grasping patterns (i.e., dial a touch-tone phone and use a spoon or fork; Table 4), the standard prosthesis performed better than the 3D printed arm. The patient reported that their concerns of durability with the 3D printed prosthesis paired with the increased dexterity of the standard prosthesis lead to the overall decreased use of the 3D printed prosthesis.

## Discussion

Only a few studies have compared standard prostheses versus 3D printed arm prostheses. Previous case studies have compared standard myoelectric arm [23] or partial hand prostheses [24] with comparable 3D printed prostheses. Lee et al. [23], compared a standard myoelectric and 3D printed pressure sensor-controlled arm prostheses in a 52-year-old male patient with a transradial traumatic amputation. In agreement with the results of the present study, the authors found increased functional performance in the Box and Block test and nine hole pegboard test using the 3D printed prosthesis. Both prostheses allowed the participant to perform activities that use gross or composite grasping, such as transferring a paper cup and putting on socks. However, for activities that require high dexterity (i.e., individual finger movement) and grip strength, the standard myoelectric prosthesis resulted in higher performance. Baun et al. [24], compared a standard myoelectric partial hand prosthesis with a body-powered 3D printed partial hand prosthesis in a 48-year-old male with partial hand amputation of the non-dominant left hand. The authors [24] reported lower performance for the 3D printed partial hand prosthesis when compared to the myoelectric prosthesis in the Southampton Hand Assessment Protocol and a lower rating in a semi-structured interview. However, the 3D printed prosthesis performed comparably to the myoelectric prosthetic in grasping large lightweight objects and performed slightly higher in a single trial of the box and blocks test. Although the comparison of a myoelectric versus body-powered partial hand prostheses [24] may lack validity, the study provides valuable information related to functional outcomes.

The potential limitations of the present investigation include learning effects, differences in device functionality and design, and conflicting survey scores. Box and blocks and bimanual coordination trials were not randomized and thus did not account for the subject learning the procedure over time. These learning effects are likely insignificant due to the limited number of trials; however, it would be unreasonable not to account for this limitation in the future to eliminate it. Stark differences in the actuation of the prosthetics created a limitation in comparative analysis. For example, a prosthetic hook's terminal device is actuated by shoulder movement. Meanwhile, pinching and grasping actions in the 3D printed prosthetic device were actuated by flexion of the elbow. Differences in terminal devices may have also impacted the comparison of functionality; the 3D device's hand versus the standard device's hook differ significantly in gripping power and versatility. Moreover, the standard prosthesis allowed for passive gripping force, as it was a voluntary-opening device. Lastly, there were conflicting results in the QUEST and OPUS survey. The durability of the 3D printed device received a 2 in the QUEST (Table 2), while durability received a 4 in the OPUS (Table 3). This could be due to the subject submitting these ratings at different times or recalling different experiences during time of submission. Additionally, user effectiveness ratings in the QUEST reported that the standard device exhibited superior performance with a rating of a 5 (very satisfied) compared to 3D printed device's rating of a 2 (not very satisfied). Similar results were reported in the OPUS survey of functional performance.

Overall, the findings from previous investigations [23, 24] and the current case study may suggest that 3D printed prostheses are equal or superior to standard prosthesis in functional activities that include gross or composite grasping, and less functional in activities that require individual finger movement, different grasping patterns, and strong gripping. Furthermore, the perceived low durability of 3D printed prostheses represents a constant concern [7, 12]. The limited grip strength could be ameliorated with the use of hybrid 3D printed prostheses which could use linear actuators that drive finger flexion and increase grasp strength in individuals with shorter residual limbs. While the 3D printed functional hands are limited in terms of grasping patterns and individual finger movement, future designs could implement more advanced terminal devices that allow for complex finger movement and varied grasping patterns, while maintaining the low-cost 3D printed proximal portion of the prosthesis. Additionally, the 3D printed transradial prosthesis requires a residual limb of at least 3–4 cm in length to appropriately flex the elbow and articulate the fingers. Individuals with shorter residual limbs can be fitted with additional thermal plastic around their limb that inserts to the 3D printed socket to allow for a longer lever arm. Nonetheless, the main advantage of a 3D printed prosthesis is the visual appeal of the device, rapid and cost-effective manufacturing method, and the ability to fit these devices remotely [7, 12]. The ability to fit a 3D printed arm prosthesis remotely has the potential of making transitional or postoperative prosthesis accessible to a large number of individuals in rural or isolated areas. Health care professionals from developed countries could remotely fit and manufacture transitional prostheses and send them to the medical team supervising the patient [7].

## Conclusion

The use of a 3D printed arm prosthesis fitted remotely resulted in better functional performance, but lower overall patient satisfaction than the standard arm prosthesis. The durability concerns and the lack of individual finger movement represent a major limitation of 3D printed prostheses. However, the positive attributes of these devices, including visual appeal, easiness to put on, reduced weight, and customization of dimensional parameters to improve upper-limb symmetry, offer promising options.

## Methods

This case report describes the functional and patient satisfaction outcomes of a 3D printed arm prosthesis fitted remotely versus a standard arm prosthesis. Furthermore, this case report also describes the process of designing, fitting, and manufacturing a 3D printed arm prosthesis. The patient was a 59-year-old male (height: 177.8 cm and weight: 81.6 kg) with a traumatic transradial amputation of the dominant arm (Fig. 1A). The residual forearm was 21 cm in length and 24.5 cm in circumference. The non-affected forearm was 28 cm in length and 28 cm in circumference. The research participant was informed about the study, and a consent form was explained and signed. The study was approved by the University of Nebraska Medical Center Institutional Review Board.

The patient visited the laboratory on two occasions. During the first visit, the patient used the standard prosthesis and hook attachment to perform the Box and Block Test to assess unimanual gross dexterity [15] and a bimanual coordination tray to assess hand synchrony. The Box and Block Test was performed using methods previously described by Mathiowetz et al. [16]. In short, the participant was asked to sit comfortably in front of a partitioned box (53.7 cm × 25.4 cm, 8.5 cm), with his hands at the sides of the box. When instructed to begin, the participant moved as many 2.5 × 2.5 × 2.5 cm blocks from one partition to the other within one minute, only moving one block at a time. Three trials were performed per hand, for a total of six trials during each visit.

The bimanual task was recorded using an experimental instrument previously described by Kilbreath et al. [17], examining inter-limb coordination in individuals who survived strokes. The bimanual coordination tray required the participant to rest his hands on the instrumented box, shoulder width apart, with his back to the chair. Both wrists were fitted with magnetic straps, which triggered electromagnetic sensors in the box, as well as the trays that are lifted during the tasks. The participant was asked to reach forward and grab both trays (hand-to-tray) and transport them 110 mm forward and 8 mm upward to the ledge on the box (tray transport). Finally, they were to return their hands to the starting position (hand return) [17]. This task was performed for three trials each visit.

**Fig. 3:**
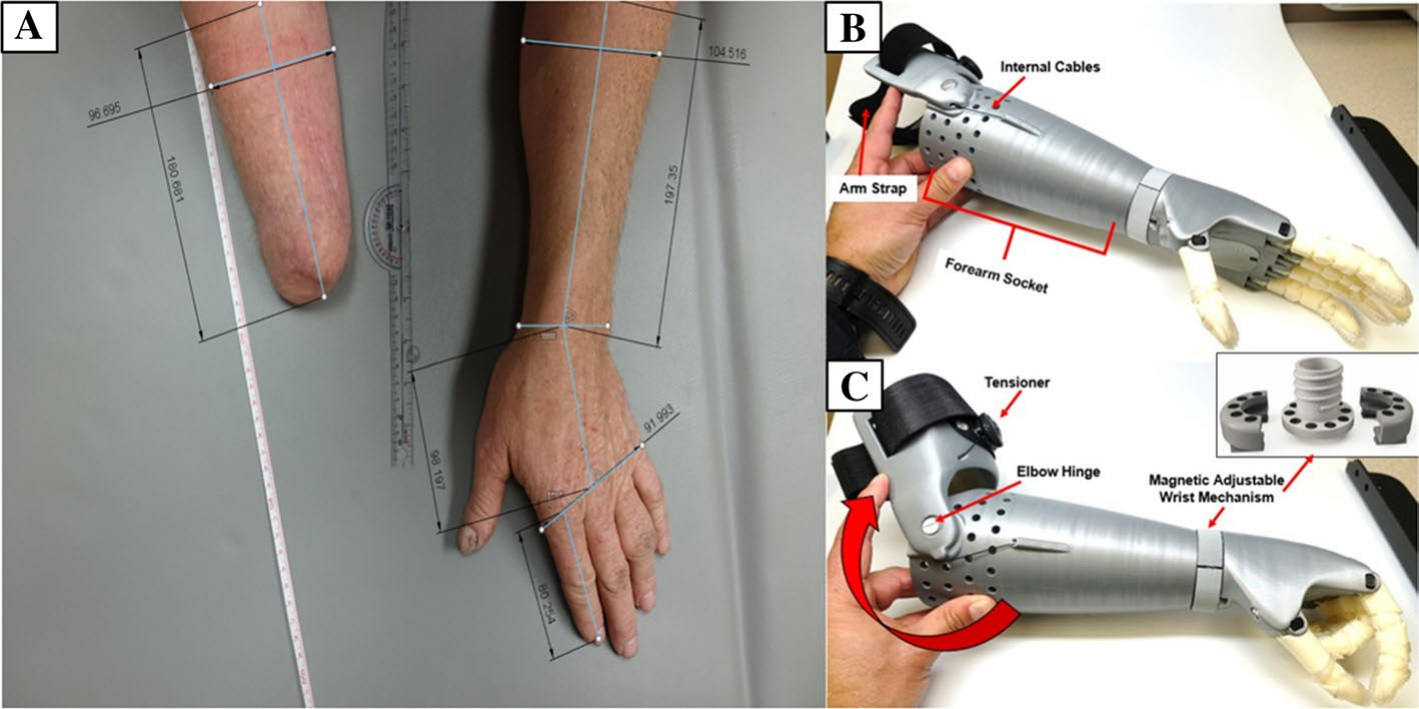
**A** Template photograph used for the remote fitting procedures of the 3D printed arm prosthesis. (1) Affected forearm width. (2) Affected forearm length. (3) Non-affected forearm width. (4) Non-affected forearm length. (5) Non-affected hand width. (6) Non-affected forearm length. Dashed red lines indicate measurement boundaries over wrist joint of the non-affected hand. **B** The transradial arm prosthesis in the open position. Elastic cords placed inside the palmar aspect of the fingers provide passive finger extension. **C** Finger flexion was activated through 10°–20° of elbow flexion of the residual functional joint in the direction of the red arrow. The wrist can be manually adjusted

The patient reported using the standard prosthesis for four to five hours a day for 5 weeks before his first laboratory visit. Measurements were verified during the second visit and the 3D printed device was shipped to the participant in 2 days. During the second visit, the patient used a 3D printed arm prosthesis (Fig. 3B, C) and performed the same testing described in the first visit. The patient reported using the 3D printed arm prosthesis 6 h per week for 5 weeks before his second laboratory visit. The patient completed functional and satisfaction surveys after using each prosthesis. Prosthesis use and satisfaction were assessed using the Quebec User Evaluation of Satisfaction with assistive Technology (QUEST 2.0) [2,^18^] and a modified version of the Orthotics Prosthetics Users Survey (OPUS) [19, 20]. The QUEST 2.0 is a 12-item measure that assesses user satisfaction with the device and service provided, and has shown to be a validate and reliable measure in other populations such as multiple sclerosis [21]. The Modified OPUS [20] is a validated instrument to specifically measure prosthetic and orthotic device outcomes. We specifically administered the upper extremity functional status survey (UEFS), as well as the OPUS Satisfaction with Devices (CSD) and Satisfaction with Services (CSS).

The 3D printed arm prosthesis was a voluntary-closing prosthesis powered by elbow flexion (Fig. 3B, C). The device was secured using a Velcro strap in the arm cuff of the 3D printed prosthesis, so no harness was required to suspend this device. Using the BOA closure system (Mid power reel M3, BOA Technology Inc., Denver, Colorado), the patient can rotate the dial on the proximal portion of the prosthesis (Fig. 3C, see “tensioner”) to increase the tension on the flexion cables that run through the forearm and to the phalanges of the prosthesis. This allows for incremental levels of elbow flexion necessary to close the hand. Magnets embedded into the wrist joint of the 3D printed arm prosthesis allowed passive adjustments of wrist rotation. Cable entanglement was prevented by a pivot system connecting the hand and the arm cables at the wrist. The hand allowed composite phalangeal flexion and index–thumb pinch grasping actions that were actuated by the elbow's flexion. Elbow flexion of 10° would produce 2.5 cm of cable travel for full operation. Silicone finger grips were added onto the fingertips to increase friction and prevent slippage of gripped objects. The prosthesis was designed to be proportional to the length and circumference of the participant's non-affected limb. Scaling the prosthesis was performed remotely and began with instructing a family member to photograph both the affected and unaffected limbs, including a known measurable scale, such as a ruler (Fig. 3A). This photo was then uploaded to Autodesk Fusion 360 and used as a backdrop. Using the ruler included in the photograph, Autodesk Fusion 360 was calibrated by informing the software the distance between two centimeter hatch marks on the ruler. This photogrammetric method allowed the extraction of several anthropometric measurements from the photographs. The measurements collected were: residual limb length and width, the non-affected limb’s forearm length and width, hand length and width, as well as finger length. Circumferences of the residual limb were estimated based on width measured. Once the prosthesis was scaled to the patient's arm and measurements were confirmed by a certified prosthetist, the files were uploaded to the 3D printer. The prosthesis was manufactured using polylactic acid. Polylactic acid was chosen as it is a low-cost material that is biodegradable and possesses thermoforming characteristics that facilitate post-processing and final adjustments. The average printing time is between 8 and 10 h using an Ultimaker 2 Extended (Ultimaker 2, Ultimaker B.V., Geldermalsen, The Netherlands). The 3D printed arm took 2 days to construct and 2 days for shipping.

The standard arm prosthesis consisted of a socket made of flexible material, a hook terminal device, a wrist unit to enable rotation of the hook, a Bowden control-cable system, and a figure-9-harness that supports the prosthesis. The prosthetic hook was a voluntary-opening device primarily controlled by flexion of the glenohumeral joint for grasping objects distant from the midline of the body (i.e., forward-reaching) and biscapular abduction to grasp objects close to the midline of the body (i.e., buttoning a shirt). The standard voluntary-opening hook required 5 cm of cable travel for full operation. The standard arm prosthesis was fitted in a clinic by a certified prosthetist. The average time of prosthetic fitting after amputation is roughly 12.7 months [22]. Specifically, our participant did not receive his hook prosthesis until 12 months after his amputation. This further highlights the expedience of the 3D printed device fitment and manufacturing, which may reduce prosthetic rejection [5, 22].

## Data Availability

The datasets used and/or analyzed during the current study are available from the corresponding author on reasonable request.

## Abbreviations

3D: Three dimensional
QUEST 2.0: Quebec User Evaluation of Satisfaction with Assistive Technology
OPUS: Orthotics Prosthetics Users Survey
OPUS—CSD: Orthotics Prosthetics User Survey—Satisfaction with Devices
OPUS—CSS: Orthotics Prosthetics User Survey—Satisfaction with Services
UEFS: Upper Extremity Functional Status Survey
